# Dutch reference values for the Patient-Reported Outcomes Measurement Information System Scale v1.2 - Global Health (PROMIS-GH)

**DOI:** 10.1186/s41687-021-00314-0

**Published:** 2021-05-12

**Authors:** Ellen B. M. Elsman, Leo D. Roorda, Martine H. P. Crins, Maarten Boers, Caroline B. Terwee

**Affiliations:** 1 Department of Epidemiology and Data Science, Amsterdam UMC, Vrije Universiteit Amsterdam, Amsterdam Public Health research institute, de Boelelaan 1089a, 1081 HV Amsterdam, the Netherlands; 2 Amsterdam Rehabilitation Research Center | Reade, Amsterdam, the Netherlands; 3 Zuyderland MC department of Quality and Safety, Amsterdam, the Netherlands; 4 Amsterdam Rheumatology and Immunology Center, Amsterdam UMC, Vrije Universiteit Amsterdam, Amsterdam, the Netherlands

**Keywords:** PROMIS, Global Health, General population reference values, Patient-reported outcome measures

## Abstract

**Background:**

To add context to the impact of medical conditions, it is important to interpret and compare health outcomes across studies and populations. We aimed to determine Dutch reference values for the Patient-Reported Outcomes Measurement Information System Scale v1.2 - Global Health (PROMIS-GH).

**Methods:**

The PROMIS-GH, also referred to as PROMIS-10, was completed by 4370 Dutch persons, representative for the 2016 Dutch population. T-scores for the mental health (GMH) and physical health (GPH) subscales, and their shorter two-item subscales, were calculated for the entire population, age groups and gender. T-scores for GMH and GPH were compared to the US reference population, representative for the 2000 US general population. Interpretability thresholds for poor, fair, good, very good and excellent GPH and GMH were calculated based on T-scores of participants, which were categorized into five groups based on their response to item Global01. For each group the mean GPH and GMH T-score was calculated and the midpoint between two adjacent means was identified, resulting in thresholds. Thresholds based on the Dutch data were compared to US thresholds.

**Results:**

The Dutch population had a GMH T-score of 44.7 and a GPH T-score of 45.2, both substantially worse than the US reference population T-score of 50. Lower T-scores were also found for age-range and gender subpopulations. Dutch GMH and GPH interpretability thresholds were mostly not substantially different compared to the US thresholds, although the Dutch threshold between fair and poor mental health was considerably higher (29 vs. 38).

**Conclusions:**

This study reports reference values for the PROMIS-GH scale for the Dutch general population, including age-range and gender subpopulations. These reference values provide an important tool for healthcare professionals and researchers to better evaluate and interpret patient-reported mental health and physical health. Scores are notably worse than the US reference values. The exact reason for this remains subject for further research, although possibilities for the differences are discussed, including the presence of differential item functioning and the representativeness and recentness of the data.

## Background

Health-related quality of life is increasingly used as an outcome measure for the effectiveness of intervention programs and the evaluation of care in the general population (for example interventions and care aimed at prevention) and in populations with specific diseases [1–3]. Generic health-related quality of life instruments are broadly applicable and can be applied to many different impairments, diseases, patients and populations, whereas disease-specific instruments are designed for specific patient populations [4]. Global health instruments evaluate the overall health status, rather than specific domains of health as evaluated by domain-specific instruments [5].

An instrument often used to assess global health is the Short Form Health Survey (SF)-12 [6]. The SF-12 consists of 12 items summarized into two subscale scores: the Physical Component Summary and the Mental Component Summary. A similar instrument was developed by the Patient-Reported Outcomes Measurement Information System (PROMIS®) initiative: the PROMIS Scale v1.2 – Global Health (PROMIS-GH) [5]. Compared to the SF-12, this scale measures related though distinct health constructs (for example fatigue vs. vitality and emotional distress vs. mental health) and has similar completion time and reliability [5]. However, in contrast to the SF-12, PROMIS-GH was developed with item response theory (IRT), which has several advantages. With IRT, items are ordered on a scale (metric) based on the item ‘difficulty’. For example, an item ‘are you able to get out of bed?’ is considered an ‘easier’ item of physical function than an item ‘are you able to walk 5 miles?’. These differences are taken into account when calculating IRT-based scores. Furthermore, several IRT models allow each item to have different discriminative ability, which means that each item has a different contribution to the reliability of a score. With IRT-based scoring a person’s score is based on the pattern of item responses, taking item parameters (difficulty and discriminative ability) into account. This means, for example, that the lowest response (‘very severe’) to the question “How would you rate your fatigue on average?” (Global08) does not get the same weight as the lowest response (‘poor’) to the question “In general, how would you rate your physical health?” (Global03). Instruments developed with classical test theory, such as the SF-12, do not take differences in item difficulty and discriminative ability into account [7, 8]. The PROMIS-GH scale represents 5 core health domains (physical function, pain, fatigue, emotional distress and social health), as well as general health, cutting across these domains [9]. The PROMIS-GH scale consists of 10 items, and is therefore sometimes referred to as PROMIS-10. Responding to global health items, respondents weigh different aspects of health, in order to arrive at a final indicator of their health status. Global health items provide an efficient assessment of self-reported health, and are predictive of for example health care use and mortality [10]. The PROMIS-GH scale can also be used to predict several preference-based index scores [11, 12], such as the EQ-5D and HUI-3, which are useful to provide information regarding the value of different health states for cost-utility analyses.

The PROMIS-GH contains 2 subscales, Global Mental Health (GMH) and Global Physical Health (GPH), each containing 4 items. Apart from the 2 4-item subscales, even shorter 2-item subscales have been developed. These subscales, referred to as the GMH-2a and GPH-2a are more feasible for use in clinical practice [13]. All subscales demonstrated sound psychometric properties [5, 13, 14]. Moreover, the subscales fitted an IRT model, enabling the calculation of IRT-based scores.

The PROMIS-GH has been translated to a variety of languages, including a Dutch-Flemish translation [15]. Psychometric evaluation of the Dutch-Flemish translated PROMIS-GH supported structural validity, internal consistency, measurement invariance and cross-cultural validity in the Dutch general population, although item Global10, from the GMH subscale, showed misfit to the IRT model, which means that the item has a lower relation with the other items in the scale than expected (lowest item-scale correlation) (Pellicciari L, Chiarotto A, Giusti E, Crins M, Roorda L, Terwee C: Psychometric properties of the Patient-Reported Outcomes Measurement Information System Scale v1.2 - Global Health (PROMIS-GH) in a Dutch general population, submitted).

The International Consortium for Health Outcomes Measurement (ICHOM) has included PROMIS-GH in the standard set for Overall Adult Health, which represents the outcomes that matter most to all adults, including those with no disease, well controlled disease and poorly controlled disease [16]. Care providers are advised to use the PROMIS-GH to better understand how to improve the lives of their users.

There is increasing interest in the interpretation and comparison of PROMIS scores across studies and populations to add context to the impact of diseases and conditions. An important feature of PROMIS measures is that scores are represented as T-scores, which are centered on the US Census population, with an average score of 50 and a standard deviation of 10 [17]. As such, a general reference frame for a person’s health status is provided relative to the US reference population [18]. However, the average health of the general population in other countries might be higher or lower than 50 [18]. The aim of this study was therefore to estimate and evaluate Dutch reference values for the PROMIS-GH.

## Methods

### Participants

Participants were selected from an existing internet panel of the Dutch general population by a data collection company (Desan Research Solutions; certified for ISO-20252 – market research and opinion research and ISO-27001 - data security). The panel was provided by Global Market Insite (GMI). On a voluntary basis, panelists were recruited mainly through telephone and ads and banners on websites. Informed consent to become a panelist is ensured by GMI. Panelists receive ‘panel points’ for participating in research, which they can collect at regular intervals to receive a small amount of money, or – more often – a web voucher. For this particular study, panelists were recruited by an invitation from the panel host to participate. The invitation indicated the topic and length of the survey, and participants received panel points equal to a small monetary incentive. By voluntarily responding to the invitation for this survey, panelists provided informed consent to participate in the study. All data collected were strictly anonymous, as the data collection company did not know the identity of the respondents, and the panel provider did not know what panelists responded to the survey. Given that the responses were strictly anonymous at all times during the study, the only possible risk for participants could have been in the survey itself. However, the senior researchers involved in this project at the data collection company and the panel provider tested and evaluated the questionnaire and did not identify any risks. Participants needed to be representative of the Dutch general population with respect to age distribution, gender, education level (low, middle, high), regions (as an indicator for urbanization; north, east and south are in general more rural, whereas west is in general more urban) and ethnicity (native, first- and second-generation western immigrant, first- and second-generation non-western immigrant). Representativeness of the participants was compared to data from Statistics Netherlands in 2016 (www.cbs.nl), with a maximum allowable deviation of 2.5% as criterion. The Medical Ethical Committee of Amsterdam UMC, location VUmc, the Netherlands, confirmed that the study protocol was exempted from ethical approval according to the Dutch Medical Research in Human Subjects Act (WMO), as no experiments were conducted. The study adhered to the tenets of the Declaration of Helsinki.

### Procedures

Data from this study was collected in 2016 as part of a larger study aimed at validating 8 full Dutch-Flemish PROMIS item banks plus the PROMIS-GH scale in the Dutch general population [19]. Participants were asked to complete the PROMIS-GH items, in addition to a block consisting of one or more full PROMIS item banks, through a web-based survey which did not allow to skip any items. Additionally, participants answered questions regarding their sociodemographic characteristics (age, gender, educational level, region of residence, ethnicity).

### PROMIS Global Health

PROMIS-GH consists of 10 items. Table 2 provides a full description of the items. The items concern general health (Global01), quality of life (Global02), physical health (Global03), mental health (Global04), social discretionary (Global05), physical function (Global06), pain (Global07), fatigue (Global08), social roles (Global09) and emotional problems (Global10) [5]. Each item is scored on a 5-point Likert scale, except Global07 which is rated on a 11-point numerical scale and then recoded to a 5-point Likert scale. For each item, a higher score represents better heath, except for items Global08 and Global10, which are therefore reversed coded when calculating a score. Scores of 2 GMH and GPH subscales can be calculated, each containing 4 items. The GMH subscale, representing mental health, consist of Global02, Global04, Global05 and Global10 [5]. The shorter GMH-2a subscale is calculated with the items Global04 and Global05 [13]. The GPH subscale, representing physical health, consist of Global03, Global06, Global07 and Global08 [5]. The shorter GPH-2a subscale is calculated with the items Global03 and Global06 [13]. The items Global01 and Global09 do not contribute to the calculation of subscale scores [5]. However, scores of these items and the other items of PROMIS-GH, can be reported individually as well.

Total scores are derived from the IRT model and expressed as T-scores, with a mean of 50 and a standard deviation of 10 for the US reference population [17]. According to PROMIS convention, all T-scores are calculated based on the item parameters from the original US calibration sample [20]. Higher scores represent better global mental/physical health. T-scores can be calculated by uploading item scores in the online HealthMeasures Scoring Service program, provided by the US Assessment Center [21] or by calculating raw sum scores and converting them to T-scores with the conversion table in the PROMIS-GH Scoring Manual [22]. Scoring Service is the most accurate scoring method because it uses IRT-based response pattern scoring, thereby taking item difficulty and discriminative ability into account, and can handle missing data (the conversion table can only be used when all items are completed) and was therefore used in this study.

### Statistical analyses

Descriptive statistics were used to summarize the sociodemographic characteristics of participants and responses to the PROMIS-GH items. T-scores for the GMH and GMH-2a, and GPH and GPH-2a were calculated for the entire population, for age groups (18–34 years, 35–44 years, 45–54 years, 55–64 years, 65–74 years and ≥ 75 years) and for gender. T-scores were compared to the US reference population and age-range and gender subpopulation reference scores of the US reference population [23].

Dutch thresholds for GPH and GMH T-scores were calculated based on T-scores of the Dutch general population with a method previously applied to the US reference population [24, 25]. That is, 1) participants were categorized into five groups based on their response to item Global01 (in general, would you say your health is excellent, very good, good, fair or poor), 2) for each group the mean T-score for GPH and GMH was calculated, and 3) the midpoint between two adjacent means was identified [24, 25]. For example, the mean GPH T-score for ‘excellent’ was 60 and for ‘very good’ was 54. The midpoint between these scores is 57, and as such the threshold for excellent physical health was set to ≥57. Likewise, the mean GPH T-score for ‘good’ was 47 and for ‘very good’ was 54. The midpoint between these scores is 51 and thus the threshold for good physical health was set to ≥51. The range for good physical health thus ranges from 51 to 56. Thresholds for GPH and GMH T-scores of the Dutch general population were visually compared to threshold available from the US reference population [25], and implications of the differences were discussed.

## Results

The PROMIS-GH was completed by 4370 participants from the Dutch general population. Table 1 shows sociodemographic characteristics of the participants. Sociodemographic differences between study participants and the Dutch general population in 2016 were all less than 2.5%. Table 2 presents the distribution of responses to the items of the PROMIS-GH.

**Table 1:** Sociodemographic characteristics of participants and the Dutch general population

Sociodemographic characteristic	Study participants^**b**^ (***n*** = 4370)	Dutch adult population 2016^**a**^ (***n*** = 13,6 million)
Age in years, mean ± SD (range)	51 ± 17 (18–93)	
18–39	33	34
40–65	44	44
> 65	23	23
Gender		
Male	47	49
Female	53	51
Educational level		
Low	29	30
Middle	41	40
High	30	30
Region of residence		
North	10	10
East	21	21
South	21	22
West	48	47
Unknown	0	
Ethnicity		
Native	78	79
1st and 2nd generation western immigrant	12	10
1st and 2nd generation non-western immigrant	10	11

*SD* standard deviation

^a^ Based on data from statistics Netherlands (https://www.cbs.nl)

^b^ All results expressed as % unless otherwise noted

**Table 2:** Distribution of responses to PROMIS-GH items

Item	Item content	Distribution of responses over the response categories (%; total ***n*** = 4370)													Mean score (SD)
		Poor (1)		Fair (2)			Good (3)		Very good (4)			Excellent (5)			
Global01	In general, would you say your health is:	7.0		30.9			41.9		16.2			4.1			2.8 (0.9)
Global02	In general, would you say your quality of life is:	4.3		24.9			44.9		21.4			4.6			3.0 (0.9)
Global03	In general, how would you rate your physical health:	8.1		33.6			38.9		15.8			3.7			2.7 (0.9)
Global04	In general, how would you rate your mental health, including your mood and your ability to think:	4.4		20.4			42.2		24.8			8.3			3.1 (1.0)
Global05	In general, how would you rate your satisfaction with your social activities and relationships:	5.8		23.1			45.0		20.9			5.2			3.0 (0.9)
		Not at all (1)		A little (2)			Moderately (3)		Mostly (4)			Completely (5)			
Global06	To what extent are you able to carry out your everyday physical activities such as walking, climbing stairs, carrying groceries or moving a chair?	2.8		9.7			18.5		17.6			51.4			4.1 (1.2)
		(worst pain imaginable)	10	9	8	7	6	5	4	3	2	1	0	(no pain)	
Global07r	In the past 7 days, how would you rate your pain on average?		0.4	0.9	4.5	10.4	9.8	8.8	6.3	9.2	11.5	13.6	24.6		3.1 (2.7)
		Very severe (1)		Severe (2)			Moderate (3)		Mild (4)			None (5)			
Global08r	In the past 7 days, how would you rate your fatigue on average?	2.7		15.6			35.5		32.3			13.9			3.4 (1.0)
		Poor (1)		Fair (2)			Good (3)		Very good (4)			Excellent (5)			
Global09r	In general, please rate how well you carry out your usual activities and roles:	4.4		23.3			45.8		21.3			5.2			3.0 (0.9)
		Always (1)		Often (2)			Sometimes (3)		Rarely (4)			Never (5)			
Global10r	In the past 7 days, how often have you been bothered by emotional problems such as feeling anxious, depressed or irritable	1.7		12.0			29.9		32.8			23.6			3.7 (1.0)

Table 3 shows the reference values of the GMH, GMH-2a, GPH and GPH-2a for the Dutch general population. T-scores on the shorter GMH-2a were comparable to the regular GMH subscale, with the largest difference being 0.5 points. Differences between T-scores on the GPH-2a and the regular GPH subscale were also mostly small (< 1), but a difference of 1.0 points was found in T-scores for participants aged 65–74 years and a difference of 1.2 points in T-scores for participants aged ≥75 years.

**Table 3:** PROMIS GMH and GPH reference values^a^ for the Dutch general population by age and gender and comparisons with the US reference population [23]

		Global Mental Health			Global Mental Health-2a	Global Physical Health			Global Physical Health-2a
	N Dutch population (%)	N US population (%)	Dutch mean T- score (SD)	US mean T-score (SD)	Dutch mean T-score (SD)	N US population (%)	Dutch mean T-score (SD)	US mean T-score (SD)	Dutch mean T-score (SD)
Total	4370 (100)	5215 (100)	44.7 (8.0)	50.0 (10.0)	44.9 (7.6)	5228 (100)	45.2 (9.2)	50.0 (10.0)	45.2 (8.3)
Gender									
Male	2069 (47)	2206 (42)	45.5 (8.0)	50.8 (10.0)	45.4 (7.5)	2212 (42)	46.1 (9.2)	51.2 (9.8)	45.6 (8.5)
Female	2301 (53)	3008 (58)	44.1 (8.0)	49.4 (10.0)	44.4 (7.6)	3015 (58)	44.5 (9.1)	49.1 (10.1)	44.9 (8.2)
Age in years									
18–34	891 (20)	1183 (23)	45.6 (8.0)	48.5 (9.7)	45.8 (7.6)	1182 (23)	47.8 (8.0)	51.6 (8.4)	48.6 (7.5)
35–44	753 (17)	863 (17)	43.8 (8.3)	48.4 (10.4)	44.0 (7.8)	865 (17)	45.2 (8.2)	50.1 (9.8)	45.7 (7.5)
45–54	646 (15)	902 (17)	43.6 (8.1)	48.2 (10.3)	43.8 (7.6)	910 (17)	44.6 (9.3)	48.2 (10.9)	44.7 (8.2)
55–64	918 (21)	873 (17)	43.6 (8.0)	50.3 (10.5)	43.8 (7.6)	875 (17)	43.4 (9.7)	48.8 (11.3)	43.3 (8.7)
65–74	893 (20)	715 (14)	45.9 (7.4)	53.1 (8.8)	45.8 (7.1)	713 (14)	45.1 (9.5)	51.0 (9.9)	44.1 (8.4)
75+	269 (6)	679 (13)	47.3 (7.7)	53.4 (8.4)	46.8 (7.4)	683 (13)	44.9 (9.8)	49.9 (9.2)	43.7 (8.7)

*SD* standard deviation

^a^ T-scores, higher scores represent better health

As shown in Table 3, the Dutch general population scored worse on mental and physical health compared to the US population. Dutch participants reported a mental health T-score of 44.7, substantially lower than the mean of T-score of 50 for the US population (mean difference − 5.3 points, 95%-CI -5.5;-5.0). The physical health T-score of participants was also lower (45.2) relative to the mean T-score of 50 for the US population (mean difference − 4.8 points, 95%-CI -5.0;-4.5). Lower T-scores for the Dutch general population were also found for age-range and gender subpopulations compared to US subpopulation reference values. T-scores of the Dutch general population showed a similar pattern compared to US reference values: males score better than females and T-scores worsen with increasing age, but improve again in the oldest age groups.

Dutch general population interpretability thresholds for GPH were similar to US reference population thresholds, as were thresholds for GMH, although the threshold for poor was substantially higher for the Dutch general population compared to the US reference population (29 vs. 38, Table 4) [25]. This would cause an increase of participants categorized as having poor mental health when thresholds for the Dutch general population would be used.

**Table 4:** Thresholds for GPH and GMH T-scores based on US reference population [25] and the Dutch general population

	NL Global Mental Health threshold	US Global Mental Health threshold	NL Global Physical Health threshold	US Global Physical Health threshold
Poor	< 38	< 29	< 35	< 35
Fair	38–42	29–39	35–43	35–41
Good	43–48	40–47	44–50	42–49
Very good	49–55	48–55	51–56	50–57
Excellent	≥56	≥56	≥57	≥58

## Discussion

Using a large representative sample, this study presents reference values for the PROMIS-GH scale for the Dutch general population. Relative to the US reference population, the Dutch general population reports worse mental and physical health. Interpretability thresholds for classification into subgroups calculated based on data of the Dutch general population did not differ much from thresholds based on the US reference population, except for the threshold for classification of poor GMH. This study also provides updated insight in the global physical and mental health of the Dutch general population, which can be used to compare mental and physical health of disease populations with a reference population.

Most mean differences between T-scores of the Dutch general population and the US reference population were around 5 points or more, both for the total population and age-range and gender subpopulations. A recent study found a within-patient difference of 2.5 points to be minimally important for the GPH subscale [26]. This is in line with minimal important difference estimates that have been determined for other PROMIS measures (between-patient differences of 2–5 points) [27–29]. This would imply that the differences in T-scores between the Dutch general population and the US reference population might be meaningful. Moreover, looking at the interpretability thresholds, a difference of 5 points often can result in being categorized into another group, which might also indicate that the differences between T-scores of the Dutch general population and the US reference population are substantial.

Dutch GMH and GPH interpretability thresholds calculated with responses to item Global01 mostly did not appear to be different compared to thresholds based on responses of the US reference population [25]. However, the threshold between fair and poor mental health was substantially higher for Dutch participants compared to the US reference population (38 vs. 29). In other words, to be categorized as having poor mental health according to the US thresholds, one has to have a relatively low mental health T-score, whereas a higher T-score suffices to be categorized as having poor mental health according to the Dutch thresholds. If the Dutch thresholds would be used for the Dutch population, more persons would be categorized as having poor mental health. One should remember that the thresholds are based on the responses of a single item on general health, which is routinely used in many settings, but not part of the GMH and GPH subscales [22]. Using a single item comes at the tradeoff of lower reliability and higher measurement error compared to the GMH and GPH subscale scores [24]. Moreover, international differences exist in the way people respond to single items on general health, as responses are influenced by norms and expectations about health of persons, groups and societies [30]. This might have contributed to the discrepancy found in the threshold between fair and poor mental health for the Dutch and US population. Thus, when using these thresholds one should consider its limitations, and bear in mind that it mainly facilitates the interpretation of PROMIS GMH and GPH scores.

The most important question evolving from the results in this paper, is whether, and if so, why the Dutch general population reports to have substantially lower GMH and GPH T-scores than the US reference population. There are several possible explanations. First, the presence of differential item functioning (DIF) might cause the Dutch population to answer items differently compared to the US population, controlling for an estimate of the measured construct. The Dutch wording of the items might have a slightly different nuance that matters in global health. However, no DIF for language was detected in the validation study of the PROMIS-GH scale in the Dutch general population (Pellicciari L, Chiarotto A, Giusti E, Crins M, Roorda L, Terwee C: Psychometric properties of the Patient-Reported Outcomes Measurement Information System Scale v1.2 - Global Health (PROMIS-GH) in a Dutch general population, submitted), using exactly the same dataset. It should be noted that DIF in that validation study was investigated using the “lordif” package, which uses an iterative hybrid approach of logistical ordinal regression and IRT [40]. Although multiple methods and software packages exist for detecting DIF [32–35], without general consensus regarding the best method, a study suggest that lordif might fall short in detecting DIF compared to for example “IRTPRO” software, which uses a two-step Wald approach [36]. Thus, there might have been more DIF than discovered in the validation study of the PROMIS-GH scale, causing or contributing to the differences in T-scores between the Dutch and US population. Since the iterative hybrid approach, used in the lordif package, is the most commonly used approach for evaluating DIF in PROMIS measures [32], it was outside the scope of the present study to further investigate DIF for language using other methods or software packages. Second, the differences in T-scores might be caused by a higher than expected proportion of participants with diseases or disabilities in the Dutch sample. Lack of data on the presence of morbidity is a major limitation of this study. Data on Years Lived with Disability (YLDs) from the Global Burden of Disease study show no evidence that the Dutch population is unhealthier than the US population [37]. In 2016, the Netherlands had 13,100 YLDs per 100,000 persons, while the US had 15,507 YLDs per 100,000 persons [37], indicating that the US has a larger burden of disease. Given that both study samples are representative for their country on other variables, there seems no reason why the Dutch respondents would report worse global health. On the other hand, one could argue that individuals who have time to participate in an online panel to complete questionnaires, might more often be persons without full-time employment, for example caused by physical or mental disability. Moreover, potentially important indicators such as income levels and employment status were not considered when creating samples, as it becomes more difficult to create representative samples when more variables are included. Third, there might be demographical differences between the Dutch and US population that could explain the differences in T-scores. The Dutch sample contained a higher proportion of males and older persons (Table 3). However, the Dutch general population still reports worse T-scores than the US reference population when matched on age or gender. Thus, differences in demographics probably do not explain the differences in T-scores found. Fourth, the data on which the centering sample of the PROMIS-GH scale is based, might be outdated, as the data was already collected in 2006–2007 [38]. A subsample representing the 2000 US census was subsequently used to center the scores [38]. Data for the current study was collected in 2016, and is representative for the Dutch population in terms of sociodemographic variables in that same year. In the PROMIS 2010 re-centering project data was collected by an internet survey company (www.op4g.com) from a convenience sample that has similar demographic characteristics as the 2010 US census. Those respondents reported worse health by about half a standard deviation compared to the original PROMIS general population sample on various item banks and the global health scale (personal communication with developers of the PROMIS-GH scale) [39]. This is comparable to the T-score differences for global health found in this study. Another study in the US general population found a mean GPH T-score of 48.3 and a GMH T-score of 48.5 [40]. It must be stressed that scores of the Dutch population on other item banks are more comparable to US reference scores. For example, a sample of the participants in this study also completed the PROMIS item banks ‘Ability to participate in social roles and activities’ and ‘Satisfaction with social roles and activities’. Their mean T-score was more comparable to the T-scores of the US reference population (50.6 and 47.5, respectively) [19]. In light of these results, the presence of additional DIF might offer a possible explanation for the differences in T-scores between the Dutch and US population on the PROMIS-GH scale, but the age of the US data and the potential non-representativeness of the Dutch sample on important indicators might also play a role. Further research is warranted to fully understand the differences.

The availability of Dutch general population reference values provides an important tool for healthcare professionals and researchers to better evaluate and interpret patient-reported mental health and physical health. The presented reference values by age and gender also allow a more tailored and relevant interpretation and understanding of T-scores within these subgroups. Incorporating these tailored reference values in the feedback patients receive on their completed PROMs, might help to provide more culturally appropriate and easier to interpret information to patients and healthcare professionals. For Dutch and Flemish users, the Dutch-Flemish Assessment Center offers real-time IRT-based scoring of the PROMIS-GH (by the same algorithm as Scoring Service) for use in clinical practice, through a software link with several data collection platforms.

This study shows that a general population outside of the US may have different mean global health scores than the US reference population that was used to define the PROMIS metric. A study using the PROMIS-29 suggested that this may also be the case in other countries [18]. We recommend to provide regularly updated country-specific reference values obtained from representative populations, in order to aid interpretation and understanding of T-scores in clinical practice and research.

## Conclusions

The Dutch population had a GMH T-score of 44.7 and a GPH T-score of 45.2, both substantially worse than the US reference population T-score of 50. Lower scores were also found for age-range and gender subpopulations. Dutch GMH and GPH interpretability thresholds were mostly not substantially different compared to the US thresholds, although the Dutch threshold between fair and poor mental health was considerably higher. The Dutch reference values provide an important tool for healthcare professionals and researchers to better evaluate and interpret patient-reported mental health and physical health. Further research is necessary to investigate the exact reason for the differences in T-scores for the Dutch and US population.

## Data Availability

The datasets used and/or analyzed during the current study are available from the corresponding author on reasonable request.

## Abbreviations

YLDs: YLDs Years Lived with Disability
DIF: Differential item functioning
IRT: Item response theory
GMH: Global mental health
GMI: Global Market Insite
GPH: Global physical health
ICHOM: International Consortium for Health Outcomes Measurement
PROMIS: Patient-Reported Outcomes Measurement Information System
PROMIS-GH: PROMIS Scale v1.2 – Global Health
SF-12: Short Form Health Survey-12
WMO: Dutch Medical Research in Human Subjects Act [Wet Medisch-Wetenschappelijk Onderzoek]
